# Recent Progress in Environmental Toxins-Induced Cardiotoxicity and Protective Potential of Natural Products

**DOI:** 10.3389/fphar.2021.699193

**Published:** 2021-07-08

**Authors:** Yuanying Yang, Shanshan Wei, Bikui Zhang, Wenqun Li

**Affiliations:** ^1^ Department of Pharmacy, The Second Xiangya Hospital, Central South University, Changsha, China; ^2^ Institute of Clinical Pharmacy, Central South University, Changsha, China

**Keywords:** environmental toxins, cadmium, arsenic, pesticides, cardiotoxicity, natural products

## Abstract

Humans are unconsciously exposed to environmental toxins including heavy metals as well as various pesticides, which have deleterious effects on human health. Accumulating studies pointed out that exposure to environmental toxins was associated with various cardiopathologic effects. This review summarizes the main mechanisms of cardiotoxicity induced by environmental toxins (cadmium, arsenic and pesticides) and discusses the potential preventive effects of natural products. These findings will provide a theoretical basis and novel agents for the prevention and treatment of environmental toxins-induced cardiotoxicity. Furthermore, the limitations of current studies, future needs and priorities are discussed.

## Introduction

The term “toxicity” refers to the relative ability of exogenous chemical substances to cause direct or indirect damage after contacting with living organisms or entering living organisms. Toxicity is closely related to dose, route of exposure, and duration of exposure. Accumulating evidence demonstrated the toxicity of drugs or other environmental factors to organs and systems. In mammals, aminoglycoside compounds are selectively toxic to renal proximal tubule cells and sensory hair cells of inner ear, resulting in nephrotoxicity and ototoxicity (Kros and Steyger, 2019). In a recent prospective study, it was found that approximately one out of 2,300 amoxicillin-clavulanic acid users experienced liver damage (Björnsson and Hoofnagle, 2016). The heavy metal lead, as a neurotoxin, has an adverse effect on human cognition and behavior (Rocha and Trujillo, 2019). Although the whole organs in the human body are vulnerable, heart as a vital organ is noteworthy.

Heart disorders such as arrhythmias and ischemic heart disease, are one of the leading causes of morbidity and mortality in the world (Al-Mallah et al., 2018). In addition to pathological factors, drug factors and environmental factors can also lead to heart disease. Cardiomyocytes are considered to be the main target of doxorubicin cardiotoxicity, and the clinical application of doxorubicin is limited by cumulative and dose-related cardiotoxicity, which may lead to congestive heart failure (Yu et al., 2018). In the past few decades, evidence on the role of exposure to environmental toxins in cardiac damage has increased rapidly.

Environmental toxins, including heavy metals, pesticides, petroleum by-products and other toxic chemicals, are not easily degraded and have a long residual period. Moreover, they can be enriched in the human body through bioconcentration and food chain, thereby causing deleterious health effects. Heavy metals refer to inorganic elements with a density greater than 5 g/cm^3^ which can be classified into two groups based on their toxicity: essential and non-essential heavy metal. 1) Essential heavy metals are harmless or relatively less harmless at low concentration (Zn, Cu, Fe, and Co). 2) Non-essential metals are highly toxic even at low concentration (Cd, Hg, As, and Cr) (Kim et al., 2019). Metalloids such as arsenic usually fall into the category of heavy metals due to its similar chemical properties and environmental behavior (Yang et al., 2018). Heavy metal contamination in water and soil has rapidly increased during the last few decades due to fossil fuel combustion (McConnell and Edwards, 2008), urban waste disposal, mining and smelting, and the application of fertilizers and pesticides (Hu and Cheng, 2013; Kapusta and Sobczyk, 2015). Recent studies have provided convincing evidence that exposure to heavy metals is associated with increased risks of diabetes and hypertension (Hu and Cheng, 2013; Planchart et al., 2018), which are strong risk factors for cardiovascular diseases. Exposure to arsenic is associated with various cardiopathologic effects. For example, arsenic exposure may cause arrhythmia by prolonging the QT interval and accelerating intracellular calcium overload (Manna et al., 2008). As a toxic heavy metal, cadmium can damage the cardiovascular system, leading to heart disorders such as myocardial infarction, cardiomyopathy, and heart failure (Mollaoglu et al., 2006; Everett and Frithsen, 2008; Peters et al., 2010).

The post World War II era witnessed a great change in agriculture practices, which led to the emergence of mechanization and the development of various chemical pesticides. Pesticides are often used to keep crops healthy and free from disease and pests. Moreover, pesticides have a wider range of uses, such as acaricides and rodenticides for non-plant use to control disease vectors. The main chemical pesticides include organochlorines, organophosphates and carbamates. With the expansion of agricultural production scale, the application of pesticides has shown a sharp upward trend. The use of pesticides has indubitably increased food production and eased the problem of famine to a certain extent, but the threat of pesticides to life and health has gradually emerged and cannot be ignored. Rare arrhythmias can appear later after organophosphorus poisoning, even if the state of illness improves markedly, which may be caused by cardiotoxic effects, metabolic acidosis and electrolyte disturbances (Georgiadis et al., 2018). The cardiotoxic effect of aluminum phosphide leads to oxidative stress, deplete myocardial energy ATP and induce apoptosis in animals (Baghaei et al., 2016). Paraquat poisoning induces alterations in myocardial function and left ventricular geometry, such as enlarged left ventricular end-systolic diameter (Wang et al., 2014).

Natural products with multiple biological activities are becoming a significant beginning of novel agents and own various pharmaceutical development potentials. This review summarizes current understanding of environmental toxins-induced cardiotoxicity and discusses the potential preventive effects of natural plants.

## Mechanisms of Environmental Toxins-Induced Cardiotoxicity

### Oxidative Stress

Oxidative stress is a typical phenomenon of many cardiovascular abnormalities, as well as the most widely studied and recognized mechanism of iatrogenic cardiotoxicity of environmental toxins. The expression and coordinated induction of antioxidant enzymes are mediated by the antioxidant response element, which is a key mechanism to resist chemically induced oxidative/electrophilic stress. Nuclear factor erythroid-2 related factor 2 (Nrf2) is an important transcription factor that regulates cellular oxidative stress, which binds to antioxidant response element sites and regulates antioxidant-mediated gene expression and induction (Jaiswal, 2004). Endogenous antioxidants including superoxide oxide dismutase (SOD), catalase (CAT) and glutathi-one peroxidase (GPx) have been identified as Nrf2-regulated antioxidant enzymes and constitute a line of defense against reactive oxygen species (ROS), which play a vital role in maintaining redox balance and protecting cells from oxidative damage. Once the production of ROS and the internal antioxidant defense system is out of balance, cells will experience oxidative stress (Das et al., 2016). A study conducted by Doroshow et al. (1980) demonstrated that the heart, compared to liver, has a much lower concentration of endogenous antioxidants, which indicates that the heart may be more sensitive to peroxidation damage due to its limited antioxidant capacity in scavenging oxygen free radicals.

Early studies have shown that heavy metal Cd promotes the release of iron in biomembrane and this free iron participates in Fenton-type reactions, thus generating ROS (Casalino et al., 1997). Another study reported that Cd may competitively bind to the q_0_ site of cytochrome b of mitochondrial complex III, leading to the accumulation of semiubiquinone at the q_0_ site, generating ROS and causing oxidative stress (Wang et al., 2004). Moreover, as a thiol affinity metal, free Cd mainly targets intracellular glutathione (GSH)—a reactive oxygen scavenger. The depletion of the GSH pool will lead to insufficient clearance of Cd, which triggers the disorder of the cell redox balance (Nair et al., 2013). The same conclusion can also be obtained in the study of the cardiotoxicity mechanism of arsenic and other heavy metals (Shimizu et al., 1998). It is speculated that the overproduction of mitochondrial reactive oxygen species (mtROS) may lead to the occurrence of various cardiovascular diseases (Li et al., 2013a). Arsenic-induced overproduction of mtROS destroys myocardial mitochondrial complex I-IV, thereby down-regulating the expression of mitochondrial respiratory chain complex (Adil et al., 2015). Earlier, it was also believed that the increased production of mtROS caused mitochondrial swelling and abnormal mitochondrial membrane potential.

Just like many drugs, lithium may directly or indirectly induce oxidative stress through mitochondria via the redox cycling or by promoting iron accumulation and oxidation/nitrification modification of essential mitochondrial proteins; in this way, it plays a key role in the development of myocardial dysfunction (Castro et al., 2011).

Fine particulate matter (PM2.5) can produce a large number of ROS and reactive nitrogen species (RNS), caused the unbalance of cell homeostasis and then excessive ROS may result in damage of nuclear DNA and mitochondria including mitochondrial permeabilization, membrane potential decreasing and mitochondrial swelling, followed by further apoptosis in cardiomyocytes (Yang et al., 2018).

It is reported that the redox state of experimental animals exposed to organophosphate is generally abnormal. Georgiadis and his colleagues pointed out that oxidative stress is the main mode of action of organophosphates, leading to side effects on myocardial tissue (Georgiadis et al., 2018). The fact that long-term exposure to organophosphorus pesticides inhibit the activity of cholinesterase, increased the production of ROS, which disrupts the balance of antioxidant enzymes, thereby leading to oxidative stress (Razavi et al., 2015; Asghari et al., 2017a). As for other pesticides, most of them will experience the same process. Phosphorus aluminum can inhibit antioxidant enzymes and iron release from transferrin, causing Fenton’s and Haber-Weiss reactions to produce iron-catalyzed ROS, thereby inducing oxidative stress (Anand et al., 2011). Paraquat can trigger a continuous redox cycle reaction, producing a large amount of ROS, leading to oxidative stress and impairing myocardial contractility, ultimately causing heart failure (Wang et al., 2017). Chlorpyrifos can cause myocardial tissue disorders, myocardial fiber degeneration and connective tissue edema. It is speculated that these changes may be due to increased secretion of mtROS and oxidative stress in heart tissue, which lead to decreased mitochondrial energy, decreased secretion of proteolytic enzymes, and DNA disintegration to induce apoptosis (Kapoor and Kakkar, 2014).

### Changes in Cardiac Ion Channels and Ion Disorders

Cardiomyocytes are rich in sodium channels, and pyrethroids are believed to be able to convert the voltage-dependent activation and inactivation of sodium channels into hyperpolarization potentials. Type I pyrethroid, tefluthrin, and type II pyrethroid, fenpropathrin and α-cypermethrin, alter the time course of sodium channel current by changing the relative ratio of fast and slow inactivation current, modify the voltage dependence of I (Na), prolong the ventricular action potential and cause post-depolarization, indicating an arrhythmogenic activity (Spencer et al., 2001). Ion content plays a role in many physiological processes. Proper ion concentration is essential to ensure the normal function of the entire body, especially the heart. Atrazine induced cardiotoxicity via the modulation of cardiac ATPase including Na^+^-K^+^-ATPase, Ca^2+^-ATPase, Mg^2+^-ATPase and changes in the transcription of pump subunits, leading to ionic disorder (Lin et al., 2016). PM2.5 significantly inhibited mitochondrial Na^+^-K^+^-ATPase and Ca^2+^-ATPase activities, implying that PM2.5 causes dysfunction of sodium pump and calcium pump (Li et al., 2015). One of the typical features of NaAsO_2_-induced cardiotoxicity is the accumulation of intracellular calcium (Chen et al., 2010). Myocardial L-type calcium channel plays a vital role in maintaining calcium ion balance. The accumulation of intracellular calcium in myocardial tissue may cause various abnormalities, including ventricular arrhythmia and systolic dysfunction (Ficker et al., 2004).

### Inflammation

Excessive toxic metals impair immune function and the accumulation of immune complexes, and lead to cardiovascular diseases through a series of interrelated processes, such as the uncontrolled release of inflammatory cytokines. Cd poisoning increases the production of TNF-α, IL-2 and IL-6 by stimulating circulating monocytes and tissue macrophages, leading to the synthesis and release of pro-inflammatory cytokines, which may be attributed to the increase in oxidized low-density lipoprotein (ox-LDL) caused by cadmium (Janabi et al., 2000). Arsenic may directly induce atherosclerosis by increasing the mRNA transcription of growth factors such as granulocyte-macrophage colony stimulating factor (GMCSF) and TGFα, and inflammatory cytokines such as TNF-α and IL-6 (Alamolhodaei et al., 2015) and multiple evidence indicate atherosclerosis is closely related to ischemic heart disease.

## Protective Effects of Natural Products Against Environmental Toxins-Induced Cardiotoxicity

### Cadmium-Induced Cardiotoxicity

Heavy mental Cadmium (Cd), a ubiquitous environmental pollutant, was listed as the first category human carcinogen by the International Agency for Research on Cancer (IARC) in 1993. Human exposure to Cd can be through many sources, including eating food contaminated by pesticides and fertilizers, smoking cigarettes, and working in cadmium-contaminated workplace, with smoking being a major risk factor (Paschal et al., 2000). Like other toxic heavy metals, Cd is greatly concentrated in the upper levels of food chain (Järup and Akesson, 2009). Its extremely long biological half-life (about 20–30 years) and low rate of excretion from the body lead to Cd continuous bioaccumulation in soft tissues (Branca et al., 2018), which inevitably induces acute and chronic tissue damage and does great harm to important organs, such as liver (Rikans and Yamano, 2000) and kidney (Satarug, 2018), even our heart (Ali et al., 2020), depending on the time and dose. It has been long realized that the toxicity induced by Cd triggers biochemical and physiological changes in the heart. A growing number of data has shown that excessive exposure to Cd is closely related to cardiovascular disease (Peters et al., 2010), including cardiac death and myocardial infarction (Everett and Frithsen, 2008), according to the epidemiological studies.

**FIGURE 1:**
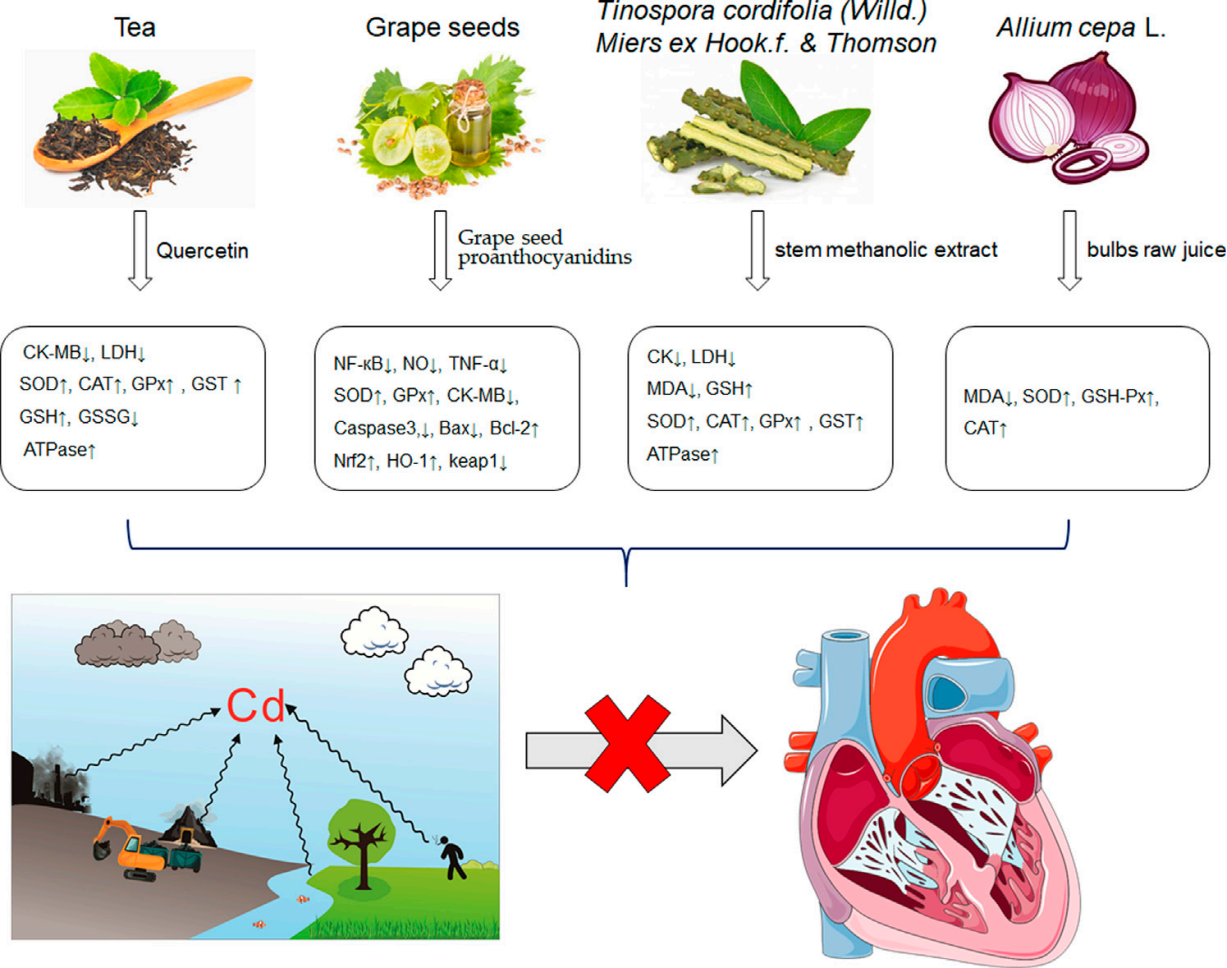
The cadmium exposure routes and protective mechanisms of natural products against cadmium-induced cardiotoxicity. CK-MB, creatine kinase-MB; LDH, Lactic dehydrogenase; SOD, Superoxide dismutase; CAT, catalase; GPx, Glutathione peroxidase; GST, Glutathione S-Transferase; GSH, glutathione; NF-кB, nuclear factor-кB; NO, nitric oxide; TNF-α, tumor necrosis factor-α; Nrf2, NF-E2-related factor 2; HO-1, heme oxygenase-1; MDA, Malondialdehyde.

It is universally acknowledged that oxidative stress is the major event induced by Cd poisoning (Liu et al., 2009). Cd exerts toxic effects via the generation of free radicals and subsequent ROS accumulation, causing tissue damage to a great extent (Gupta et al., 2017). Given the correlation between Cd exposure and oxidative stress, attention turned to focus on natural products rich in antioxidants in order to inhibit the cardiotoxicity mediated by Cd. Documented probable cadmium exposure routes and mechanisms of natural products against Cd-induced cardiotoxicity are presented in Figure 1.

#### Natural Products

Accumulating studies has proved the beneficial effects of natural products for its protection against Cd induced toxicity. Quercetin is known as robust antioxidant and free radical scavenger against oxidative stress (Xu et al., 2019). A study conducted by Milton Prabu et al. evaluated the effects of quercetin on cardiac marker enzymes, lipid peroxidation products, lipid profile, membrane bound ATPases and antioxidant status in Cd-intoxicated rats, which demonstrated that quercetin pretreatment at a dose of 50 mg/kg body mass (bm) per day could prevent oxidative damage to the rat hearts when supplemented with Cd (5 mg/kg bm/day) for 28 days (Milton Prabu et al., 2013).

Grape seed proanthocyanidins, effective bioactive components extracted from natural grape seeds, have drawn a great slew of attention due to their extensive biological and pharmacological properties (Bors et al., 2001; Bagchi et al., 2002). In Cd-administered (CdCl_2_, 5 mg/kg bm, 4 weeks) male Wistar rats, grape seed proanthocyanidins (100 mg/kg bm, p. o.) was found to own cardioprotective effects by functioning as *in-vivo* antioxidant and was capable of inhibiting the membrane disturbances in cardiomyocytes, apoptotic pathway and inflammation. Besides, the signals from phenolic antioxidants present in grape seed proanthocyanidins lead to phosphorylation of Nrf2 and/or redox modulation of Nrf2/Keap1, leading to separation of Nrf2 from Nrf2/Keap1, thereby restoring Nrf2 expression in the heart of rats. Therefore, grape seed proanthocyanidins recuperated the Cd-induced oxidative stress mediated cardiac dysfunction (Nazimabashir et al., 2015).

#### Plant Extracts

*Tinospora cordifolia* (Willd.) Miers ex Hook. f. & Thomson, belonging to the family *Menispermaceae* Juss., is a medicinal herb used in Ayurveda for treating varied metabolic disorders and toxic conditions (Patgiri et al., 2014; Singh et al., 2015). Antioxidant and cardioprotective activity of *Tinospora cordifolia* (Willd.) Miers ex Hook. f. & Thomson against streptozotocin induced diabetic rats has been reported (Sharma et al., 2011). Moreover, its stem methanolic extract for male albino Wistar rats can significantly attenuate Cd-induced lipid peroxidation and protein carbonylation, reduced heart histological changes and decreased the activities of membrane bound ATPases at dose of 5 mg Cd/kg bm (Priya et al., 2017).

*Allium cepa* L. (Onion), one of the most widely and commonly consumed vegetables in the genus *Allium* L., shows considerable antioxidant value (Suru, 2008). Onion consumption correlates with low rates of coronary heart disease (Hertog et al., 1993). Several studies have demonstrated cardioprotective effects of *A. cepa* extract by lowering serum cholesterol and blood pressure. In Cd-administered (1 mg/kg bm, s. c.) male Sprague-Dawley rats, *A. cepa* extract effectively attenuated Cd-induced histological alterations, apoptosis in the cardiomyocytes and decreased levels of the enzymatic antioxidants probably via its antioxidant and anti-apoptotic activity (Alpsoy et al., 2014).

Summarized above the mentioned, although the natural products supplementation has a protective effect on Cd-induced oxidative heart damage in rats, the exact molecular mechanism has not been elucidated. Moreover, with the exception of *in-vivo* studies, the protective activity of these natural products in Cd-induced cardiotoxicity should be verified through *in-vitro* studies.

### Arsenic-Induced Cardiotoxicity

**FIGURE 2:**
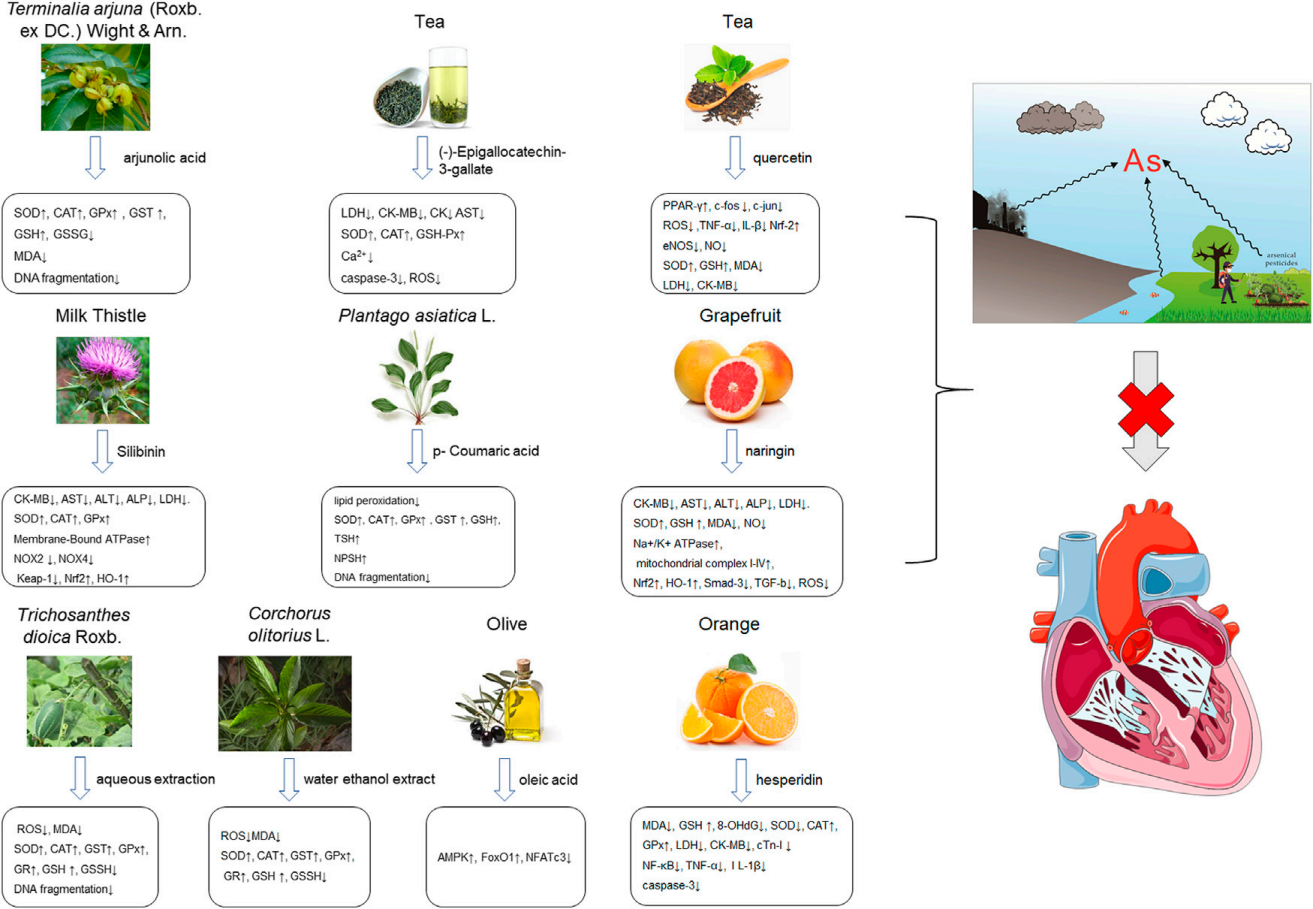
The arsenic exposure routes and protective mechanisms of natural products against arsenic-induced cardiotoxicity. AST, Aspartate transaminase; ALT, alanine aminotransferase; ROS, reactive oxygen species; IL-β, interleukin-β; eNOS, endothelial NOS; NOX2, NADPH oxidase two; NOX4, NADPH oxidase four; TSH, total sulfhydryl; NPSH, non-protein sulfhydryl; AMPK, adenosine monophosphate-activated protein kinase; GR, glutathione reductase.

Arsenic is naturally occurring metalloid element that abundant in Earth’s crust and biosphere. Due to its high water-solubility, the main source of human exposure to arsenic is drinking water contaminated with arsenic (Mathews et al., 2013). In addition, the use of arsenic-containing herbicides, pesticides, and burning fossil fuels can cause a high risk of arsenic poisoning (Mandal et al., 2001; Mazumder et al., 2010; Shivakumar et al., 2014). Once absorbed, arsenic is redistributed to almost the entire body organ system, including the heart (Ratnaike, 2003; Manna et al., 2008). Arsenic exposure gives rise to myocardial damage, arrhythmia (Manna et al., 2008) and left ventricular hypertrophy (Pichler et al., 2019), which correlates with pathogenesis of myocardial tissue. The possible mechanisms of arsenic-induced cardiotoxicity mainly include oxidative stress, DNA fragmentation, changes in cardiac ion channels (Alamolhodaei et al., 2015). At present, there are various drugs for the treatment of the above-mentioned arsenic-induced heart diseases, such as commonly used angiotensin-converting enzyme inhibitors and β-blockers (Curigliano et al., 2016). However, the non-negligible side effects limit their clinical application. In recent years, more and more attention has been focused on the chemical components extracted from natural products due to their broad pharmacological activity and low toxicity. Scientists hope to find inspiration from it and explore new methods for the treatment of arsenic-induced cardiotoxicity. As is depicted in Figure 2, the possible arsenic exposure routes and mechanism of natural products against cadmium-induced cardiotoxicity have been summarized.

#### Natural Products

Arjunolic acid, one of the constituents existed in the bark of *Terminalia arjuna* (Roxb. ex DC.) Wight & Arn., has been shown to provide significant cardioprotection in isoproterenol-induced myocardial necrosis (Sumitra et al., 2001). Manna et al. mentioned that arsenic poisoning (10 mg/kg bm, p. o., 2 days) can cause severe oxidative damage to the heart tissue in mice, which can be prevented by treating with arjunolic acid (20 mg/kg bm, 4 days). The overall preventive effect of arjunolic acid may be due to its scavenging free radicals and metal chelating properties, thereby reducing the arsenic load in the cells (Manna et al., 2008). Another research revealed that arjunolic acid could regulate oxidative phosphorylation in the mitochondria and subsequently inhibit ROS generation through abrogated p47phox-Ser345 phos phorylation in stimulated and myocardial infarction neutrophils, which is the first report showing the protective effect of arjunolic acid on p47phox phosphorylation and mitochondrial bioenergetics (Miriyala et al., 2015). Moreover, arjunolic acid as a Peroxisome Proliferator-Activated Receptorα (PPARα) agonist up-regulates PPARα, leading to repression of TGF-β signaling, especially by inhibiting TGF-β activated kinase1phosphorylation, which reduces the activity of p38MAPK and NFκBp65, ultimately reversing hypertrophy-related myocardial fibrosis. These results provide new insights for in-depth exploration of the protective mechanism of arjunolic acid against arsenic-induced cardiotoxicity.

(-)-Epigallocatechin-3-gallate, the most abundant and active compound in green tea, possesses a potent antioxidant capacity and exhibits versatile pharmacological activities (Chakrawarti et al., 2016). Treatment with (-)-epigallocatechin-3-gallate (50 mg/kg bm, i. g., 30 days) significantly attenuated myocardial injury, suppressed oxidative damage and myocardial apoptosis induced by arsenic in rats (50 mg/kg bm, 30 days) and H9c2 cells (1 µM). This study proved that (-)-epigallocatechin-3-gallate plays a protective role by reducing the production of ROS, maintaining the intracellular calcium ion concentration, and reducing activation of caspase-3 (Sun et al., 2016). The protective effect of (-)-epigallocatechin-3-gallate on arsenic-induced cardiotoxicity was evaluated *in vivo* and *in vitro*, and the mechanism may be attributable to its potent antioxidant capacity.

The plant of *Madhuca indica* J. F. Gmel., belonging to the family *Sapotaceae* Juss., commonly known as Mahua (Mohod et al., 2016), possesses a robust inhibitory effect on oxidative stress and inflammation. Its bioactive component extraction—quercetin has been proven to own the potential to fight against cardiotoxicity induced by isoproterenol and ischemia/reperfusion injury (Li et al., 2013b; Chen et al., 2013). Mukherjee et al. found that administration of quercetin (10 and 20 mg/kg bm, p. o.) showed a significant protective effect against arsenic-induced (5 mg/kg bm, 28 days) oxido-nitrosative stress and myocardial injury by regulating Nrf2, PPAR-γ, and apoptosis (c-fos and c-jun) (Mukherjee et al., 2017).

Silibinin is a polyphenolic flavonoid extracted from the seeds of milk thistle (Verdura et al., 2018), possessing strong antioxidant and cardioprotective activities (Sheta et al., 2020). It has been successfully employed as a protective agent against arsenic-induced *in vivo* model of hepatotoxicity (Muthumani and Prabu, 2012). Pre-administration of silibinin (75 mg/kg bm, 28 days) in arsenic-intoxicated (5 mg/kg bm, p. o., 28 days) rats shows potent protective efficacy against oxidative stress and cardiac injury. The treatment of silibinin facilitates the restoration of antioxidant status and normal histological architecture of cardiac tissue by inhibiting the induction of prooxidants (e.g., NOX2 and NOX4) and enhancing antioxidants (Muthumani and Prabu, 2014).

p- Coumaric acid is a naturally occurring hydroxycinnamic acid derivative which is widely found in fruits and vegetables as a dietary polyphenol. According to Prasanna et al. (2013), *p*-coumaric acid has a protective effect similar to the standard antioxidant vitamin C on the rat heart damage caused by the pretreatment of sodium arsenite (5 mg/kg bm, 30 days) with respect to oxidative stress and cardiac tissue markers due to its antioxidant properties. Subsequent experiments detected changes in the mRNA expression profiles of inflammatory cytokines, transcription factors, MAP kinases and apoptotic proteins in myocardial tissue. The results show that daily oral *p*-coumaric acid (75 and 100 mg/kg bm, 30 days) before sodium arsenate exposure can regulate the changes in the above-mentioned mRNA expression profile (Prasanna and Rasool, 2014). However, further studies should be performed to identify the exact mechanism on the protective effects of *p*-coumaric acid against arsenic toxicity.

Oleic acid, a natural triterpene compound mainly found in olive oil, has been shown to have therapeutic potential for improving pathological conditions including CVDs (Kris-Etherton, 1999). Samanta et al. pointed out that arsenic can cause cardiac hypertrophy in both mice and rat H9c2 cardiomyocytes (Samanta et al., 2020). Interestingly, they observed that oleic acid (100 µM) reduced the expression of NFATc3 by activating AMPK and increasing the localization of FoxO, thereby helping to relieve cardiac hypertrophy induced by arsenic (1 µM) in H9c2 cells. Oleic acid (12.5 mg/kg bm, 14 days) has also been observed to help improve cardiac hypertrophy in arsenic-exposed mice (arsenic trioxide, 4 mg/kg bm, i. p., at every alternate 2 days for 6 weeks). AMPK-FoxO1-NFATc3 pathway acting in arsenic-mediated cardiac hypertrophy is a novel finding, which contributes to find out novel avenues to treat the condition.

Ellagic acid, a dimeric derivative of gallic acid, is a natural polyphenol component widely found in various fruits and nuts. Ellagic acid treatment (30 mg/kg bm, 14 days) could reduce oxidative stress by reducing the levels of MDA and NO and enhancing the activity of endogenous antioxidant enzymes such as CAT, SOD, GPx, thereby effectively alleviating the cardiotoxicity induced by arsenic (10 mg/kg bm, p. o., 21 days) (Goudarzi et al., 2018). However, further research is needed to accurately understand the underlying cellular mechanisms.

Naringin is a flavanone glycoside with antioxidant, anti-inflammatory and anti-apoptotic efficacy, mainly found in citrus fruits, especially in the peels of tangerines, sweet oranges and lemons. Naringin (40 and 80 mg/kg, p. o., 28 days) can reverse the significant changes in electrocardiogram, hemodynamics and left ventricular contractile function caused by chronic administration of sodium arsenite (5 mg/kg bm, p. o., 28 days). It was testified that naringin ameliorates arsenite-induced cardiotoxicity and hyperlipidemia via regulation of TGF-b/Smad-3 and Nrf-2/HO-1 pathways along with a reduction in myocardial apoptosis (Adil et al., 2016). Hesperidin, an isomer of naringin, similarly shows a robust protective potential against sodium arsenite-induced (10 mg/kg bm, 15 days) cardiotoxicity by reducing oxidative stress, apoptosis and preventing inflammation (Kuzu et al., 2021).

#### Plant Extracts

*Trichosanthes dioica* Roxb., commonly known as parwal, is mainly cultivated as a vegetable distributed in India (Kumar et al., 2012). *T. dioica* root has been reported to possess some pharmacological properties, including anti-inflammatory and antioxidant activity (Bhattacharya et al., 2011; Bhattacharya and Haldar, 2012). Bhattacharya et al. indicated that *T. dioica* root treatment prior to arsenic intoxication (10 mg/kg bm, p. o., 8 days) possessed remarkable alleviative effect against arsenic-induced cardiotoxicity in Wistar albino rats, as evidenced by significant prevention of alterations in body weight, heart weight, and hematological and serum biochemical parameters (Bhattacharya and Haldar, 2013). In addition, *T. dioica* root treatment significantly modulated all of the myocardial antioxidative parameters and reduced DNA fragmentation. The experiment also proved that at higher doses, the effect of *T. dioica* root is comparable to that of ascorbic acid and quercetin. It is speculated that the active ingredient may be cucurbit type triterpene aglycone. The alleviative effect of *T. dioica* root against arsenic-induced (10 mg/kg, p. o., 8 days) cardiotoxicity is also reflected in aqueous extract of its fruit (Bhattacharya et al., 2014).

*Corchorus olitorius* L. belongs to the family *Malvaceae* Juss. Its leaves and tender stems are rich in several phenolic antioxidative compounds (Ndlovu et al., 2020). According to Das et al., oral administration of aqueous extract of *C. olitorius* leaves prior to NaAsO_2_-intoxication significantly protected cardiac tissue against arsenic-induced (10 mg/kg bm, p. o., 10 days) oxidative impairment and prevented hyperlipidemia and DNA fragmentation (Das et al., 2010). The cardioprotective effect may be due to the presence of substantial quantity of phytophenolics and flavonoids in the extract.

In addition to looking for new agents with the potential to combat environmental toxin-induced cardiotoxicity, the current focus has shifted to determine crucial signaling molecules/pathways in environmental toxin-induced cardiotoxicity. The up-regulation of pro-apoptotic proteins Bax, caspase-3, c-fos and c-jun and the down-regulation of anti-apoptotic protein TGF-β and Bcl-2 are involved in arsenic-induced cardiomyocyte apoptosis. Studies have demonstrated that arsenic-induced elevated oxidative stress is associated with alteration in the Nrf-2/HO-1 pathway. Besides, Arsenic-induced cardiac hypertrophy is mediated by reducing the expression of AMPK and FoxO1, thereby increasing the expression of NFATc3. Collectively, studying the signaling pathways acting in arsenic-induced cardiotoxicity will help to find novel therapeutic avenues. However, the exact molecular target of natural products remained unclear. Trace its root, lack of knockdown or overexpression methods to verify the aforementioned signaling molecules, and even no gene knockout animal models are used to evaluate the cardioprotective activity of natural products.

### Pesticide-Induced Cardiotoxicity

**FIGURE 3:**
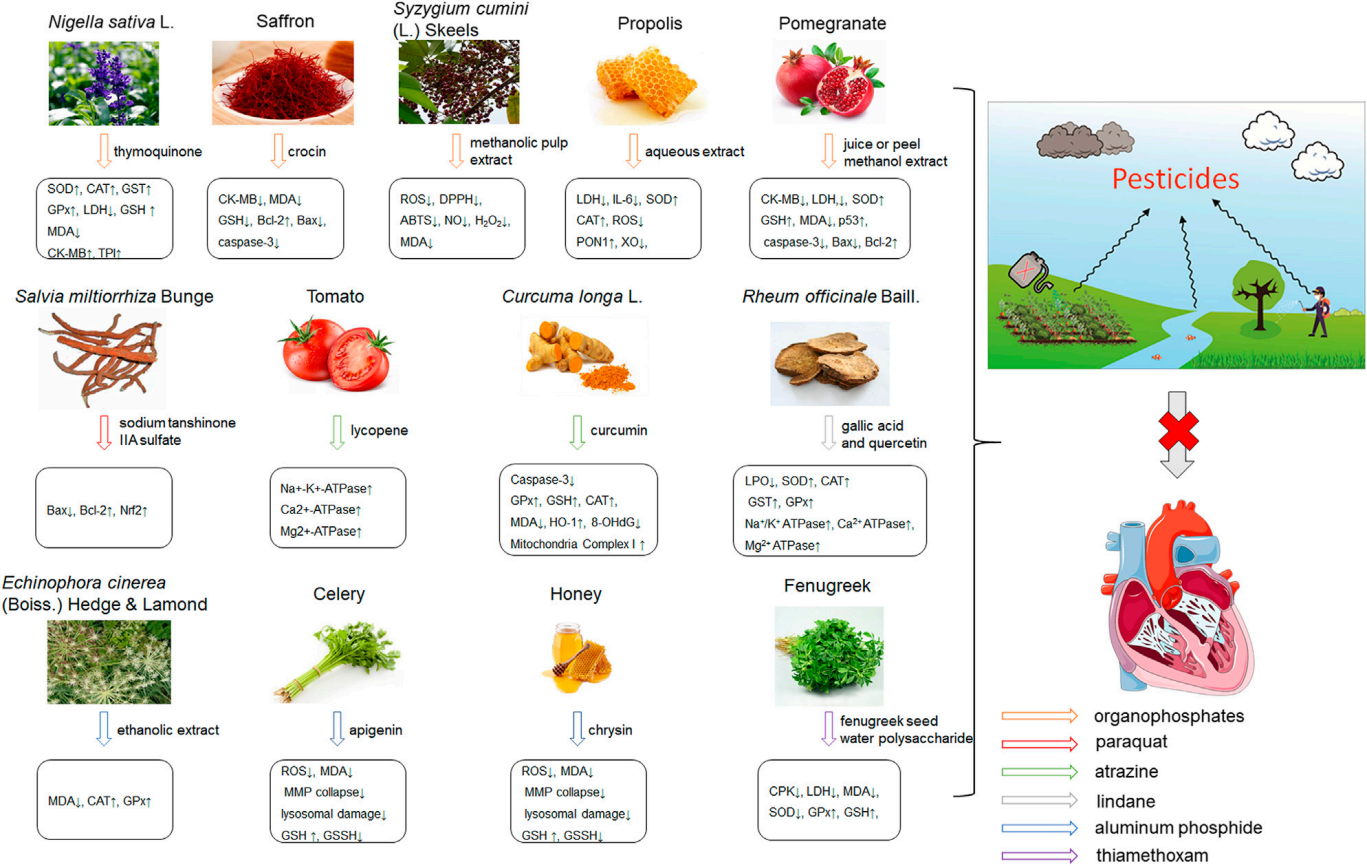
The pesticides exposure routes and protective mechanisms of natural products against pesticides-induced cardiotoxicity. LPO, Lipid Peroxide; TPI, troponin; 8-OhdG, 8-hydroxydeoxyguanosine; PON1, paraoxonase-1.

Pesticides is one of the agricultural chemicals with the largest consumption and the most abundant categories, which play a vital role in increasing agricultural production and solving human food problems. However, the use of pesticides inevitably becomes an important factor in environmental pollution. Pesticides are inherently toxic to living organisms, so its adverse effects on humans and other organisms are inevitable. A large amount of epidemiological and experimental evidence shows that there is a connection between exposure to pesticides and the incidence of multiple human diseases (Mostafalou, 2012). Exposure to pesticides has been associated with several cardiovascular complications including electrocardiogram abnormalities, myocardial infarction, functional remodeling, histopathological insults, such as hemorrhage, vacuolization, signs of apoptosis and degeneration. In addition, it also includes increased systemic and cardiac-tissue-specific oxidative stress and DNA alterations in cardiac cells that could lead to functional impairment (Georgiadis et al., 2018). Early studies have shown that pesticide chemicals may induce oxidative stress, leading to the production of free radicals and alterations in the antioxidant or oxygen free radical scavenging enzyme system (Banerjee et al., 1999). Growing evidence show that oxidative stress is a main apoptosis stimulating factor in different diseases including cardiovascular diseases and ROS induces apoptosis. Hence, this process may be inhibited by abundant natural antioxidants. A summary of possible pesticides exposure routes and mechanisms of natural products against pesticides-induced cardiotoxicity is presented in Figure 3.

#### Herbicides-Induced Cardiotoxicity

Atrazine is an extensively used herbicide of the triazine class. Due to its soil leaching properties, atrazine is easy to be leached by rainwater, irrigation water to deeper soil, or enters rivers and lakes with surface runoff. In addition, atrazine remains in surface water and groundwater for a long time and is difficult to degrade, thus causing serious pollution to the ecological environment. Exposure to atrazine can cause health risks to humans and other animal species. As early as the 1990s, atrazine and its anabolic metabolites (for example, atrazine-decrystallized-desubstituent) have been detected in heart tissue (du Preez and van Vuren, 1992) and cause cardiovascular disease during poisoning intoxication (Chan et al., 2007; Li et al., 2018). Curcumin is a polyphenol compound isolated from *Curcuma longa* L. (turmeric), which has been widely used in the food industry as a common natural pigment for a long time. Curcumin’s powerful anti-inflammatory and antioxidant effects, coupled with its ability to improve endothelial function, can reduce the risk of heart disease (Kocaadam and Şanlier, 2017). Keshk et al. concluded that the administration of curcumin (400 mg/kg bm, 21 days) can improve the cardiotoxicity induced by ATR via regulating redox state, mitochondrial function and caspase-3 expression (Keshk et al., 2014). Lycopene, a carotenoid existed in plant foods, has a higher content in ripe red plant fruits, especially in tomatoes, carrots, watermelons. With robust free radical scavenging and antioxidant ability, lycopene has attracted considerable attention as a potential chemopreventive agent against diseases such as CVD (Grabowska et al., 2019). Atrazine induced cardiotoxicity by regulating the activity of cardiac ATPase and the transcription of its subunits, thereby triggering ion disorders, while supplemented lycopene (5 mg/kg bm, 21 days) in mice significantly counteracted atrazine-induced cardiotoxicity by regulating ATPase activity and subunit transcription. Therefore, lycopene shows a significant chemopreventive potential for atrazine-induced cardiotoxicity (Lin et al., 2016).

Paraquat is a quick-acting contact-killing herbicide which has been widely used due to its good weeding effect and low environmental pollution. However, in the past few decades, there have been many deaths of paraquat, mainly due to accidental or voluntary ingestion. Once ingested orally, paraquat has extremely strong toxic effects on multiple organs, causing lung, heart, liver and kidney failure and even death (Dinis-Oliveira et al., 2008; Gawarammana and Buckley, 2011; Zhang et al., 2018). Because no effective treatment has been developed so far, the paraquat poisoning incident is extremely distressing but powerless. Medicinal plants provide abundant resources for screening new therapeutic agents. Tanshinone IIA, one of the active components of Danshen (*Salvia miltiorrhiza* Bunge), has a wide range of powerful pharmacological effects, including antioxidant and anti-inflammatory effects (Feng et al., 2019). As a clinical formulation of Tanshinone IIA, sodium tanshinone IIA sulfate is commonly used to improve myocardial blood and oxygen supply in clinical practice. According to Zhang et al., sodium tanshinone IIA sulfate therapeutically inhibits paraquat-induced myocardial cell apoptosis in rats via the enhancement of Bcl-2, the inhibition of Bax and modulating the Nrf2 pathway (Zhang et al., 2019).

#### Insecticide-Induced Cardiotoxicity

##### Organophosphates-Induced Cardiotoxicity

Approximately 40% of all pesticides commercially produced and used are organophosphates, which are widely used in the agricultural field (Kaushal et al., 2021). Organophosphates induced toxicity is mainly via inhibition of carboxyl ester hydrolases, particularly acetylcholinesterase, and it is reported that the most significant side effect of this mode of action is the oxidative stress induced in the myocardial tissue (Georgiadis et al., 2018).

Malathion is a low-toxic organophosphorus pesticide. Atale et al. (2014) conducted cell proliferation studies in malathion-treated cells, using various natural and synthetic antioxidants, including butylated hydroxyltoluene, trolox, quercetin, (−)-epicatechin, ascorbic acid, curcumin, and gallic acid. Among all the antioxidants mentioned above, gallic acid showed the most significant protection against oxidative stress. *Syzygium cumini* (L.) Skeels is a traditional medicinal plant with various bioactive compounds distributed in all parts of the plant (Chhikara et al., 2018). *S. cumini* methanolic pulp extract, rich in gallic acid, was taken to study its effect on malathion-induced toxicity. The result showed that with the treatment with *S. cumini* methanolic pulp extract, malathion-induced morphological changes, ROS levels and nuclear deformities have decreased. Increment in collagen content could also be clearly observed. Hence, *S. cumini* methanolic pulp extract could ameliorate the oxidative stress in H9c2 cells caused by malathion, which indicates that it has antioxidant properties and protective effects on malathion-induced cardiotoxicity.

Chlorpyrifos is a moderately toxic organophosphate insecticide, which was once banned from being used on vegetables due to excessive residues. The acute toxicity of chlorpyrifos mostly affects the nervous system and cardiovascular system (Cunha et al., 2018; Shou et al., 2019). It is said that chlorpyrifos poisoning induces heart tissue to produce abundant free radicals, making it particularly vulnerable to oxidative stress and peroxidative damage. As a traditional Chinese medicine, saffron (*Crocus sativus* L.) has been used for centuries around the world, known for its extensive pharmacological activities including antioxidant. The efficacy of saffron is mainly attributed to the presence of crocin (Javandoost et al., 2017), which can effectively prevent cardiotoxicity, hepatotoxicity and DNA damage (Mohammadi et al., 2018). Studies have shown that crocin can improve the histopathological changes caused by doxorubicin (Razmaraii et al., 2016), and attenuated the cardiotoxicity induced by diazinon (Razavi et al., 2013). Khalaf et al. proved that the cardiotoxicity of chlorpyrifos is mainly due to oxidative stress and crocin can alleviate this toxic effect by its antioxidant property (Khalaf and El-Mansy, 2019). Propolis, a colloidal natural product with a variety of pharmacological activities, has been widely used in medicine, health food, cosmetics and other fields. Propolis contains a lot of flavonoids and polyphenols which correlates with its physiological activity, such as anti-inflammatory, antioxidant and immunomodulatory activities (Zabaiou et al., 2017). A recent study has shown that propolis intake may reduce the risk of cardiovascular diseases caused by chlorpyrifos exposure by the improvement of PON1 and XO mRNA genes regulation leading to the accumulation of the cellular enzymatic and/or non-enzymatic antioxidants (Ibrahim et al., 2019). Pomegranate (*Punica granatum* L.) is a common fruit rich in polyphenols such as ellagic acid and ellagitannin. Administration of either its juice or peel methanol extract has shown protective potential against chlorpyrifos-induced cardiotoxicity potentially via its antioxidant, antiapoptotic and membrane stabilizing properties (El-Wakf et al., 2018).

Diazinon is a moderately toxic, novel, broad-spectrum organophosphorus pesticide. Subchronic exposure to diazinon can induce mitochondrial-mediated apoptosis in rat cardiac tissue. The above-mentioned crocin can also play a protective role in diazinon-induced cardiotoxicity by reducing lipid peroxidation and alleviating apoptosis (Razavi et al., 2013). *Nigella sativa* L. is a member of family *Ranunculaceae* Juss. Its seeds, the main source of the active ingredients of the plant, have long been used as a treatment for various pathological diseases in the Middle East and Far East (Ali and Blunden, 2003). As a strong antioxidant and anti-inflammatory agent, thymoquinone has proved to be the most bioactive ingredient in *N. sativa* seeds. Sub-acute exposure to diazinon significantly induces oxidative damage and inhibits the antioxidant defense system. Thymoquinone supplement could inhibit diazinon-induced cardiotoxicity and improved cholinesterase activity in rats via the mechanism of free radical scavenging (Danaei et al., 2019).

##### Other Insecticide-Induced Cardiotoxicity

Aluminum phosphide (ALP) is one of the most widely used metal phosphides, often used as a solid fumigant to exterminate pests (Hosseini et al., 2020). Once ALP is exposed to water or acid, it will release highly toxic phosphine gas (Kariman et al., 2012). Hence, whether it is taken orally or inhaled phosphine gas, it may cause severe poisoning or even death. Due to the extreme toxicity of ALP, more than 70% of people exposed die from its detrimental effects on various organs of the body and their main cause is cardiotoxicity. ALP-induced cardiovascular disturbances include refractory hypotension, dysrhythmia, and congestive heart failure (Asghari et al., 2017b). The primary mechanism of aluminum phosphide poisoning is the inhibitory effect of phosphine on cytochrome C oxidase (Nath et al., 2011; Mehrpour et al., 2019). Other mechanisms refer to oxidative stress, calcium and magnesium complexation, and damage to the electron transport chain (Nath et al., 2011; Anand et al., 2013). Increasing studies have shown that the active compounds found in plants are effective against chemical and drug-induced cardiotoxicity. Chrysin is a flavonoid compound with a wide range of pharmacological activities. Recently, a study in animal model showed the protective effect of chrysin against doxorubicin-induced cardiomyopathy (Mantawy et al., 2017). The protective effect of chrysin on doxorubicin-induced cardiotoxicity is exerted via the mitochondrial apoptosis pathway and the inhibition of oxidative stress (Samarghandian et al., 2017). Similarly, Khezri et al. found that treatment with chrysin significantly reduced the formation of ROS, lysosomal damage, mitochondrial damage and lipid peroxidation induced by ALP (Khezri et al., 2020). A recent study conducted by Jahedsani et al. showed that apigenin also has the same protective effect as chrysin against ALP-induced myocardial damage (Mahajan et al., 2017). Since these studies were performed in primary rat cardiomyocytes or isolated mitochondria, further studies are needed to verify the cardio-protective effects by animal trials and clinical trials on humans. *Echinophora cinerea* (Boiss.) Hedge & Lamond is a native Iranian plant, whose erial parts are often used as food seasoning in cheese and yogurt. Research suggests that a flavonoid glucoside extracted from *E. cinerea* showed cytoprotective effect against oxidative stress induced by hydrogen peroxide in PC12 cells (Shokoohinia et al., 2015). Haydari et al. demonstrated that administration of the *E. cinerea* extract can improve bradycardia, hypotension, and conduction disturbances of the rats heart caused by ALP poisoning (Haydari et al., 2020). It can also increase the level of antioxidant enzymes and protect human body from ALP-induced oxidative damage which shows a dose-dependent characteristic to a certain extent.

Lindane, an artificial chlorinated hydrocarbon pesticide which was first introduced as a scabicide for human use in the 1950s (Solomon et al., 1977), was once widely used for agricultural and public health pest control. Its persistence in the environment, high toxicity to mammals, and resistance to biodegradation have led many developed and developing countries to ban or restrict its use. As early as 2005, it has reported that lindane could cause oxidative stress, lipid peroxidation and changes in the levels of enzymatic antioxidants in the rat heart (Ananya et al., 2005). Padma et al. found that oral administration of gallic acid or quercetin protects against lindane-induced myocardial damage, possibly via maintaining the level of endogenous antioxidant enzymes and membrane-bound ATPase activity, and inhibiting lipid peroxidation (Vijaya Padma et al., 2013).

Thiamethoxam, the second-generation nicotinic insecticide, is widely used to protect cotton, sorghum and other crops from being attacked by insects and pests. Due to its slow metabolism in plants and soil and high solubility in water, thiamethoxam poses a major risk to aquatic ecosystems and human health. Feki et al. used H9c2 cardiomyoblastes and *in vivo* using Wistar rat model to confirm the toxic effect of thiamethoxam on the heart for the first time. Fenugreek (*Trigonella foenum-graecum* L.), an annual herb of the family *Fabaceae* Lindl., is considered to be one of the oldest medicinal plants. Feki et al. extracted a polysaccharide from fenugreek seeds, called fenugreek seed water polysaccharide. Administration of this polysaccharide to thiamethoxam-treated rats shows a strong protective effect on the oxidative stress of the heart, which is manifested in the significant improvement of enzymatic and non-enzymatic antioxidants and the restoration of histopathological changes in the heart tissue (Feki et al., 2019). Fenugreek seed water polysaccharide owns a great cardioprotective potential, but its specific mechanism needs to be further elucidated.

Throughout these studies mentioned above, we found that most studies on the protective effects of natural products in pesticides-induced cardiotoxicity were carried out in animals. However, *in-vitro* studies are indispensable for in-depth study of the action mechanism and complex signal networks of natural products. Due to differences in the metabolism and physiology of animals and humans, the animal models used cannot fully simulate the development of human diseases. Therefore, further clinical trials are needed to evaluate effectiveness and safety of natural products.

## Future Needs and Priorities

People are exposed to environmental toxins in various ways unconsciously. Once the environmental toxins enter the human body, they are difficult to eliminate and the content in the body slowly accumulates over time, eventually reaching the cumulative dose, damaging tissues and even organs. Although few specific environmental toxins directly target the heart, their more or less toxic effects on the heart cannot be ignored. Regrettably, despite the existence of common cardioprotective drugs in the pharmaceutical market, there is a lack of specific targeted treatment. Cardiotoxicity is still a major medical problem. Therefore, there is an urgent need to study and determine the potential molecular mechanisms and signal transduction pathways that antagonize environmental toxins-induced cardiotoxicity. Early studies mostly revealed the important role of oxidative stress, and the corresponding antagonistic method is to remove ROS. As a recent research report, the strong antioxidant resveratrol significantly inhibits the production of ROS, thereby inhibiting oxidative stress, and thus plays a protective effect on PM2.5-induced heart defects in zebrafish embryos (Ren et al., 2020). Although this method has been successful in cells and experimental animal models to some extent, the use of ROS scavengers (such as N-Acetyl-L-cysteine and L-carnitine) does not seem to be popular in the clinical practice, which indicated that oxidative stress is not the only cause of cardiotoxicity. Growing studies provided potent evidence that besides oxidative stress, environmental toxins-induced cardiotoxicity can also be affected by inflammation and changes in cardiac ion channels and ion disorders. Most natural products have multiple "targets" and may affect more than one signaling pathway. The development of multi-site targeted natural products and multi-component traditional Chinese herbal medicine can be used for the treatment of cardiotoxicity induced by environmental toxins.

The occurrence and development of cardiotoxicity are accompanied by the damage and even the death of cardiomyocytes, indicating that cardiomyocytes death may be the main cause of cardiotoxicity. The mechanism of environmental toxins-induced cardiotoxicity is sophisticated, but existing research point out that its ultimate result is cardiomyocyte apoptosis. In addition to apoptosis, a common form of cell death, drug-induced cardiomyocytes death forms also include autophagy, necrosis and other regulated forms, such as necroptosis, pryoptosis, and ferroptosis (Ma et al., 2020). Hence, whether there are other forms of cell death in cardiotoxicity induced by environmental toxins remains to be further studied.

As mentioned above, it can be found that the active ingredients in daily food, such as lycopene in tomatoes and ellagic acid in nuts and fruits, have a beneficial effect on the cardiotoxicity induced by environmental toxins, which suggests that people, especially workers who are occupationally exposed to these environmental toxins may be able to reduce the risk of poisoning by consciously increasing their intake of these foods. Epidemiological studies are needed for further confirmed.

Although in pre-clinical studies, natural products have made significant progress in preventing cardiotoxicity caused by environmental toxins, they have not yet been converted into clinical use. The main hindrance to the development of natural products-based cardioprotective adjuvants attributes to the low bioavailability of natural products (Novelle et al., 2015). Actually, natural products must be supplied in sufficient doses to enter the systemic circulation in the form of their natural structures or metabolites and reach target tissues to exert biological activity. As typical natural products with strong antioxidant capacity, polyphenols have effective protective effects on cardiotoxicity induced by environmental toxins. The antioxidant activity of polyphenols depends on their ability to scavenge free radicals, donate hydrogen atoms or electrons, or chelate metal cations, and the structure is a key determinant of their free radical scavenging and metal chelating activity (Yordi et al., 2012). However, due to low bioavailability and kinetic limitations, the direct antioxidant activity of polyphenols seems to be insufficient in the body. As Visioli et al. reported, the concentrations of polyphenols used in *in-vitro* studies usually range from μmol/L to mmol/L, while plasma metabolite concentrations rarely exceed nmol/L after normal dietary intake (Visioli et al., 2011). Although polyphenols have been shown to have indirect antioxidant capacity by regulating gene expression and endogenous antioxidant enzyme defense systems as described in our review, it is limited. Therefore, strategies such as structural modification of natural products and changing dosage forms are needed to improve the bioavailability of natural products.

Environmental toxins-induced cardiotoxicity models are mostly established on animals, primary cultured cells or cell lines. A suitable experimental model that simulates the physiology of the human heart is needed. Several natural products have been shown to have cardioprotective effects on environmental toxins-induced cardiotoxicity both *in vitro* and *in vivo*, but they are not thorough enough, and mechanisms of action have not been fully elucidated. Moreover, most of the existing research focuses on oxidation, inflammation and apoptosis, and more attention should be paid to the complex signal network, which is the core of cardiomyocyte survival and dysfunction (Ghigo et al., 2016). However, we have to admit that *in-vivo* and *in-vitro* studies have certain limitations. One of the disadvantages of *in-vitro* studies is that the effective dose used is much higher than concentration that can be achieved in humans. In addition, *in-vitro* studies use prototype compounds of natural products, rarely involving their active metabolites. As for *in-vivo* studies, due to differences in metabolism, genomics and physiology, the animal models used do not fully mimic the development of human diseases. The results obtained in *in-vivo* studies need to be transformed into humans. Collectively, a large number of compounds are proven to be effective *in vivo* and *in vitro* studies, but most of them cannot be converted into evidence for therapeutic benefits (Heinrich et al., 2020). From the discovery of new agents to the conversion into clinical use, there is still a long way to go to develop natural products to combat cardiotoxicity induced by environmental toxins.

## Conclusion

When evaluating the toxicity of environmental toxins, cardiotoxicity is an important consideration because myocardial damage can be irreversible and fatal. Mechanism of environmental toxins-induced cardiotoxicity is sophisticated and involves multiple targets and pathways, making it difficult to take corresponding intervention measures. This article reviews the mechanism of environmental toxins-induced cardiotoxicity and summarizes the protective effects of natural products on environmental toxins-induced cardiotoxicity. A list of valuable natural products has been mentioned in Table 1. Medicinal plants can be used as a source of useful novel compounds to develop effective treatments to combat cardiotoxicity in patients exposed to environmental toxins.

**TABLE 1:** Protective effect of natural products against environmental toxins-induced cardiotoxicity.

Environmental-toxins	Natural products	Structure or effective component	Model	Effect	Ref
**Cadmium**	Quercetin	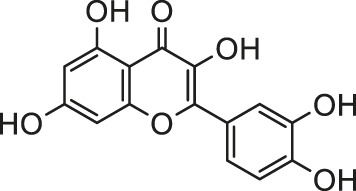	Male albino rats	Attenuated oxidative stress mediated cardiotoxicity and dyslipidemia	Milton Prabu et al. (2013)
	Grape seed proanthocyanidins	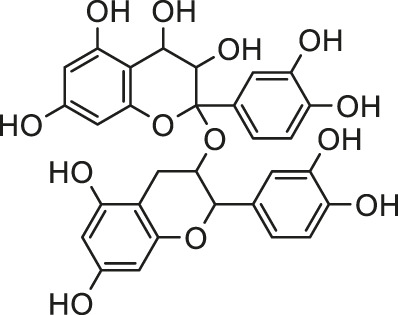	Male Wistar rats	Inhibiting the membrane disturbances in cardiomyocytes, apoptotic pathway and inflammation	Nazimabashir et al. (2015)
	*Tinospora cordifolia* (Willd.) Hook.f. & Thomson	Stem hydroalcoholic extract	Male Wistar rats	Inhibited oxidative damage, reduced heart histological alterations and decreased the activities of membrane bound ATPases	Priya et al. (2017)
	*Allium cepa* L.	Onion bulbs raw juice	Male SD rats	Reduced oxidative stress, histological damage and apoptosis	Alpsoy et al. (2014)
**Arsenic**	Arjunolic acid	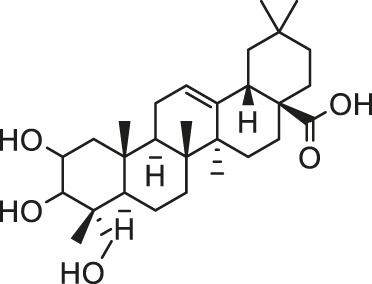	Male Swiss albino mice	Prevent cardiac oxidative impairment and maintain the normal ultra structure	Manna et al. (2008)
	(-)-Epigallocatechin-3-gallate	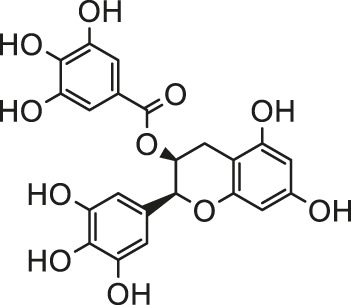	Male SD rats; H9c2 cells	Attenuated myocardial injury, oxidative damage and myocardial apoptosis	Sun et al. (2016)
	Quercetin	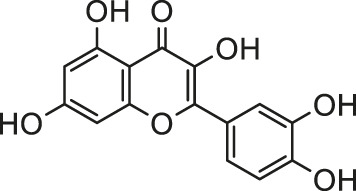	Male SD rats	Ameliorated oxido-nitrosative stress and myocardial injury	Mukherjee et al. (2017)
	Silibinin	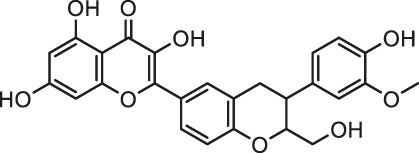	Male albino Wistar rats	Eliminated myocardial damages, facilitated the restoration of antioxidant status and normal histological architecture of cardiac tissue	Muthumani and Prabu (2014)
	p-Coumaric acid	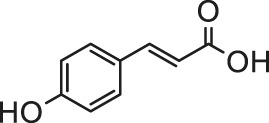	Male Wistar albino rats	Reduced myocardial oxidative stress and ameliorated cardiac damage	Prasanna et al. (2013)
	Oleic acid	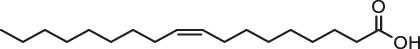	Male Swiss albino mice; H9c2 cells	Attenuated cardiac hypertrophy	Samanta et al. (2020)
	Ellagic acid	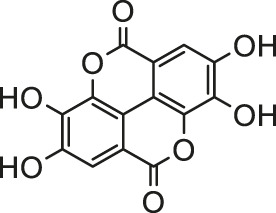	Male Wistar rats	Prevented histopathological alterations and reduced oxidative stress	Goudarzi et al. (2018)
	*Trichosanthes dioica* Roxb.	Aqueous extraction	Male Wistar albino rats	Ameliorated myocardial injury and reduced DNA fragmentation	Bhattacharya et al. (2014)
	*Corchorus olitorius* L.	Dry water ethanol extract	Male Wistar albino rats	Reduced oxidative impairment and prevented hyperlipidemia and DNA fragmentation.	Das et al. (2010)
	Naringin	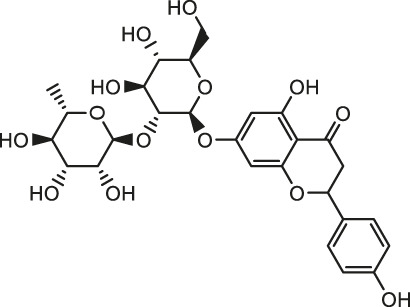	Male SD rats	Reduced ROS production and oxidative stress, scavenged free radicals, inhibited apoptosis and restored the respiratory chain Complexes	Adil et al. (2016)
	Hesperidin	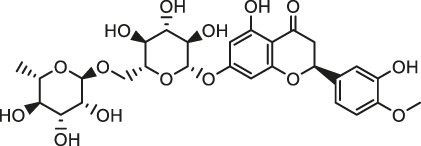	Male SD rats	Reduced oxidative stress and apoptosis, increased antioxidant enzyme activities, and prevented inflamma-tion	Kuzu et al. (2021)
Pesticides	Curcumin	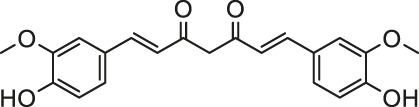	Male albino rats	Ameliorated disturbed redox status and mitochondrial dysfunction	Keshk et al. (2014)
	Lycopene	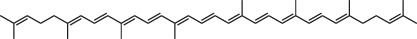	Male KunMing mice	Ameliorated cardiomyocyte ATPase function and ionic levels	Lin et al. (2016)
	Sodium tanshinone IIA sulfate	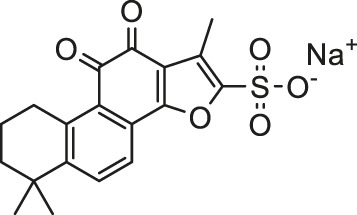	SD rats	Inhibited apoptosis, myocardial damage and failure	Zhang et al. (2019)
	*Syzygium cumini* (L.) Skeels	Methanolic pulp extract	H9c2 cells	Suppress changes in cell morphology, ROS production and ECM remodeling	Atale et al. (2014)
	Crocin	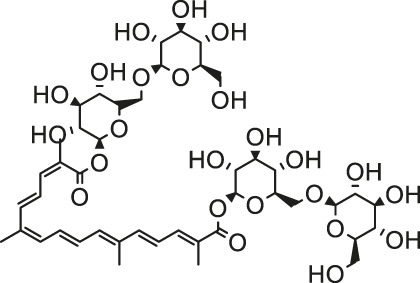	Male albino rats/male Wistar rats	Alleviated oxidative stress and ameliorated myocardial injury/reduced lipid peroxidation and alleviated apoptosis	(Razavi et al., 2013; Khalaf and El-Mansy, 2019)
	Propolis	Aqueous extract	Male albino rats	Restored antioxidant defense system disorder	Ibrahim et al. (2019)
	Pomegranate (*Punica granatum* L.)	Juice or peel methanol extract	Male albino rats	Attenuated myocardial injury and apoptosis	El-Wakf et al. (2018)
	Thymoquinone	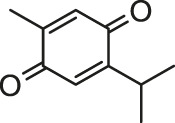	Rats (detailed strain is not mentioned)	Decreased cardiotoxicity and improved cholinesterase activity	Danaei et al. (2019)
	Chrysin	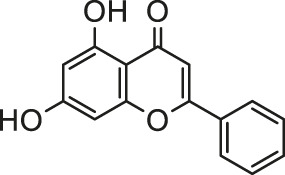	Primary male Wistar rats cardiomyocytes; isolated mitochondria	Ameliorated oxidative stress and mitochondrial damages	Khezri et al. (2020)
	Apigenin	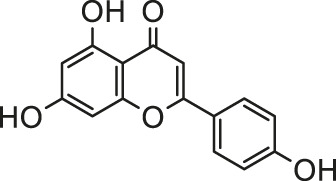	Primary male Wistar rats cardiomyocytes	Decreased cytotoxicity, oxidative, lysosomal and mitochondrial damages	Mahajan et al. (2017)
	*Echinophora cinerea* (Boiss.) Hedge & Lamond	Ethanolic extract	Male wistar rats	Improve bradycardia, hypotension, and conduction disturbances	Haydari et al. (2020)
	Gallic acid	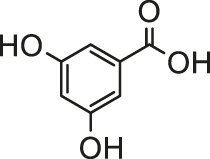	Male albino Wistar rats	Improved the status of enzymatic and non-enzymatic antioxidants and ameliorated myocardial damage	Vijaya Padma et al. (2013)
	Fenugreek (*Trigonella foenum-graecum* L.)	a polysaccharide extracted from fenugreek seeds	Male Wistar rats; H9c2 cells	Alleviated heart oxidative damage and genotoxicity	Feki et al. (2019)
